# Multiple Events Lead to Dendritic Spine Loss in Triple Transgenic Alzheimer's Disease Mice

**DOI:** 10.1371/journal.pone.0015477

**Published:** 2010-11-16

**Authors:** Tobias Bittner, Martin Fuhrmann, Steffen Burgold, Simon M. Ochs, Nadine Hoffmann, Gerda Mitteregger, Hans Kretzschmar, Frank M. LaFerla, Jochen Herms

**Affiliations:** 1 Center of Neuropathology and Prion Research, Ludwig Maximilians University, Munich, Germany; 2 Department of Neurobiology and Behavior, University of California Irvine, Irvine, California, United States of America; University of Muenster, Germany

## Abstract

The pathology of Alzheimer's disease (AD) is characterized by the accumulation of amyloid-β (Aβ) peptide, hyperphosphorylated tau protein, neuronal death, and synaptic loss. By means of long-term two-photon *in vivo* imaging and confocal imaging, we characterized the spatio-temporal pattern of dendritic spine loss for the first time in 3xTg-AD mice. These mice exhibit an early loss of layer III neurons at 4 months of age, at a time when only soluble Aβ is abundant. Later on, dendritic spines are lost around amyloid plaques once they appear at 13 months of age. At the same age, we observed spine loss also in areas apart from amyloid plaques. This plaque independent spine loss manifests exclusively at dystrophic dendrites that accumulate both soluble Aβ and hyperphosphorylated tau intracellularly. Collectively, our data shows that three spatio-temporally independent events contribute to a net loss of dendritic spines. These events coincided either with the occurrence of intracellular soluble or extracellular fibrillar Aβ alone, or the combination of intracellular soluble Aβ and hyperphosphorylated tau.

## Introduction

Alzheimer's disease (AD) is the most common age-related neurodegenerative disorder. Pathognomonic features include the accumulation of amyloid-β (Aβ) peptide, hyperphosphorylated tau protein, neuronal death, and synaptic loss [1], [2]. AD is clinically characterized by a gradual and global decline of cognitive function. Synaptic loss as one of the hallmarks of AD is the best correlate of cognitive decline and has previously been detected in the human cortex and hippocampus [1], [3]–[9]. This suggests that synaptic loss represents a critical event in the pathophysiology of AD.

It is widely believed that the abundance of Aβ plays a central role in the pathogenesis of AD. However, which Aβ species, i.e. soluble forms like monomers and oligomers or insoluble fibrils primarily contributes to AD pathogenesis and in what way remains controversial [10], [11]. Indeed, the abundance of soluble Aβ levels in the cortex correlates positively with the first cognitive deficits and synaptic loss [12], [13] long before fibrillar Aβ plaques accumulate [1], [14]. This has been modeled in AD transgenic mice that exhibit early synaptic loss and cognitive decline at a time, when only soluble Aβ is present, but before amyloid plaques become abundant [15]–[20]. Moreover, several *in vitro* studies have convincingly demonstrated that high levels of soluble Aβ directly lead to synaptic loss [21]–[25]. In addition to soluble Aβ, there is also evidence from transgenic AD mouse models that synaptic loss occurs in close proximity to insoluble fibrillar Aβ plaque deposits [26]–[31]. Besides the accumulation of soluble and insoluble Aβ, the intraneuronal abundance of hyperphosphorylated filamentous tau protein represents the second major pathological signature of AD [32]–[34], which correlates well with cognitive impairment [35], [36]. Collectively, it is of great significance to understand how soluble Aβ, insoluble amyloid plaques and hyperphosphorylated tau accumulation contribute to synaptic loss in AD.

We monitored dendritic spines by long-term two-photon *in vivo* imaging in AD transgenic mice that were crossed with YFP-H mice expressing yellow fluorescent protein (YFP) in a subset of cortical neurons [37]. Long-term two-photon *in vivo* imaging provides a powerful tool to explore structural plasticity at the level of individual spines and neurons followed over extended time periods in living mice [38]–[45]. Specifically, dendritic spines of apical dendrites of layer III and layer V neurons in the somatosensory cortex were repeatedly analyzed. Dendritic spines embody the post-synaptic side of excitatory synapses and are as such a reliable indicator of synapses themselves. They are also highly plastic structures and represent a structural correlate of learning and memory processes in the mammalian brain [46]–[48].

The aim of our study was to analyze structural plasticity of dendritic spines in triple transgenic AD mice (3xTg-AD) which progressively develop both Aβ and tau pathology in the cortex and hippocampus [49]. This leads to a distinct spatio-temporal pattern of dendritic spine loss which we characterize here for the first time.

## Materials and Methods

### Transgenic mice

Homozygous triple transgenic mice (3xTg-AD) [49] were crossed with heterozygous mice of the YFP-H line [37] (The Jackson Laboratory, Bar Harbor, USA). The offspring was crossed back with homozygous 3xTg-AD mice to yield quadruple transgenic animals homozygous for the knock-in mutation and the AD transgenes and heterozygous for YFP-H. As controls, age-matched heterozygous YFP-H mice on the same background were used. Mice were of mixed gender. All procedures were in accordance with an animal protocol approved by the University of Munich and the government of upper Bavaria (Az. 55.2-1.54-2531-110-06).

### Cranial window surgery

A cranial window over the right cortical hemisphere was surgically implanted as previously described [43], [45]. Imaging began following a 21 day rest period after surgery.

### Long-term two-photon *in vivo* imaging

Long-term two-photon imaging was performed as previously described [43], [45]. Less than 50 mW laser power was used to avoid laser-induced phototoxicity. For overview images z-stacks of 150 µm depth with 2 µm z-resolution and 1024×1024 pixels per image frame (0.22 µm/pixel) were taken with a Zeiss 40x water immersion objective (0.8 NA) to count dystrophic dendrites. In order to count dendritic spines, higher resolution images were taken with the same objective with 1 µm z-resolution and 512×512 pixels per image frame (0.11 µm/pixel). To follow the fate of individual cortical layer III neurons a Zeiss 20x water-immersion objective (1.0 NA) was used to acquire image stacks of 300 µm depth with 3 µm z-resolution and 1024×1024 pixels per image frame (0.41 µm/pixel).

### Immunohistochemistry

An immunohistochemical staining protocol was adapted from Gogolla et al. [50]: Following transcardial perfusion with 4% paraformaldehyde 100 µm thick free-floating sections were prepared. The following antibodies conjugated to biotin were used in 1∶100 dilutions: 6E10 mAb (Covance Research Products Inc., Denver, PN, USA), anti-Aβ-oligomer A11 (Invitrogen, Carlsbad, CA, USA), anti-human Tau clone HT7 mAb and anti-PHF-Tau clone AT8 mAb (Thermo Scientific Pierce Protein Research Products, Rockford, IL, USA). Secondary detection was performed with the TSA™ kit #26 with streptavidin-HRP (Invitrogen, Carlsbad, CA, USA). Alexa Fluor 647 tyramide was incubated for 3 hours. Staining of fibrillar Aβ plaques and neurofibrillary tangles was performed with 145 µM methoxy-X04 in PBS for 30 min and subsequently washed with PBS. Fluorescence images were acquired with a confocal laser scanning microscope mounted on an inverted microscope support (LSM 510 and AxioVert 200, Carl Zeiss MicroImaging GmbH, Jena, Germany). Two different immersion oil objectives were used, a 25x (LD LCI Plan-Apochromat NA 0.8) and a 40x (Plan-Apochromat NA 1.3) (Carl Zeiss MicroImaging GmbH, Jena, Germany). XYZ-spacing was 0.18×0.18×1 µm^3^ and 0.11×0.11×1 µm^3^, respectively.

### DAPI and Nissl staining

This was previously described [45]. Briefly, brains were fixed with 4% PFA in PBS at 4°C overnight. DAPI and Nissl staining was performed on 100 µm thick fixed brain slices that were cut in the same orientation as the *in vivo* images were taken. Free-floating sections were permeabilized with 2% Triton X-100 overnight. DAPI (10 µg/µl) and Nissl (20x dilution in PBS; # N-21482; Molecular Probes/Invitrogen, Carlsbad, CA, USA) was administrated and incubated for 2 hours and rinsed with PBS three times for 10 minutes. Fluorescence images were acquired with a confocal laser scanning microscope mounted on an inverted microscope support (LSM 510 and AxioVert 200, Carl Zeiss MicroImaging GmbH, Jena, Germany). Three different lasers were used for excitation: Ar ion laser at 514 nm for YFP, HeNe laser at 543 nm for Nissl, and a Ti-Sapphire laser (Mai Tai DeepSee, Spectra-Physics Lasers Division, Newport Corporation, Mountain View, CA, USA) at 780 nm for DAPI. A 25x (LD LCI Plan-Apochromat NA 0.8) immersion oil objective was used (Carl Zeiss MicroImaging GmbH, Jena, Germany). XYZ-spacing was 0.36×0.36×2 µm^3^.

### Image processing and data analysis

All images were deconvolved using the adaptive blind 3D deconvolution algorithm of AutoDeblur with 10 iterations (Version x2.0.1, Media Cybernetics Inc., Bethesda, MD, USA). The images were maximum intensity projected (Imaris 6.1, Bitplane, Zürich, Switzerland). In some figures, distracting neighboring dendritic elements were removed. The volume of dystrophic dendrites was automatically calculated using Imaris software (Version 6.1, Bitplane, Zürich, Switzerland). In this case dendrites were classified as dystrophic if they exhibited at least a 2-fold increased dendritic volume over the whole imaging period. Spines were counted in *z*-stacks by manually scrolling through the images of subsequent time points of the same position. The spine scoring method has previously described [42], [43], [51]. Spine densities refer to the amount of spines per dendrite length in µm from which they protrude. Spines lasting for less than 8 days were classified as transient and spines with a lifetime of 8 days or more as persistent [41], [52]. All results are reported as mean ±SEM. Statistical differences in measurements over time were determined using repeated measures ANOVA while statistical comparison between groups was performed with the Wilcoxon Rank Sum Test or the Student's t-test as indicated. The Pearson product-moment correlation coefficient R was calculated to determine a correlation between dendritic volume and dendritic spine density.

## Results

The 3xTg-AD mouse line harbors a knock-in mutation for presenilin 1 (PS1_M146V_) and transgenes for the amyloid precursor protein (APP_swe_) and for tau (tau_P301L_) and progressively develops both an Aβ and tau pathology in the cortex and hippocampus [49]. Soluble Aβ can already be detected at 4 months of age in the hippocampus and cortex (Fig. S1A), but fibrillar amyloid plaques do not appear before 12 months of age (Fig. S1B). Once they form, amyloid plaques are present in the hippocampus and in the frontal cortex, while the somatosensory cortex is free of plaques, even at 20 months of age (Fig. S1B). Hyperphosphorylated tau is abundant in the hippocampus and cortex from 12 months on (Fig. S1C).

### Dendritic spine loss in the hippocampus

**Figure 1:**
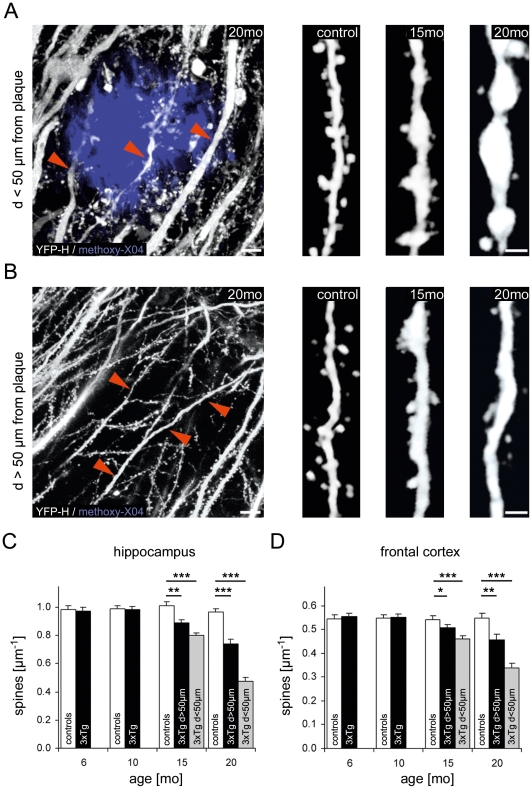
Dendritic spine density is reduced in the hippocampus and frontal cortex from 15 months on. (A, B) High-resolution images of dendrites and dendritic spines in the vicinity of (d<50 µm) and distant to (d>50 µm) amyloid plaques in the hippocampus. Scale bars: 10 µm (overviews); 2 µm (close ups) (C, D) In the hippocampus (C) and frontal cortex (D) of 15 and 20 month-old 3xTg-AD mice, the dendritic spine density was significantly reduced in areas close to (grey columns) and distant from (black columns) amyloid plaques compared to non-AD transgenic control mice (white) (* P<0.05, ** P<0.01, *** P<0.001, Student's t-test, n = 50 dendrites in n = 5 mice per group). The dendritic spine density was unchanged at 6 and 10 months of age. Error bars indicate +S.E.M.

Based on this spatio-temporal pattern of Aβ and tau pathology, dendritic spine density was analyzed in the hippocampus and frontal cortex of 6, 10, 15, and 20 month old mice by confocal imaging in fixed slices (Fig. 1). In total, 21,220 spines were counted on 50 dendrites per group (n = 5 mice per group). Hereby, we detected no difference in dendritic spine density between 3xTg-AD mice and controls at 6 and 10 months of age. This age is prior to the appearance of amyloid plaques and hyperphosphorylated tau (Fig. S1B and S1C). In 15 months old 3xTg-AD mice, once amyloid plaques and hyperphosphorylated tau are abundant, the dendritic spine density was significantly reduced not only around amyloid plaques (d<50 µm) (Fig. 1A, 1C, and 1D; hippocampus: 0.80±0.02 µm^−1^ vs. 1.01±0.03 µm^−1^, P<0.001; frontal cortex: 0.46±0.01 µm^−1^ vs. 0.54±0.02 µm^−1^, p<0.001) but also in areas distant to plaques (d>50 µm) compared to controls (Fig. 1B, 1C, and 1D; hippocampus: 0.89±0.02 µm^−1^ vs. 1.01±0.03 µm^−1^, p<0.01; frontal cortex: 0.51±0.01 µm^−1^ vs. 0.54±0.02 µm^−1^, P<0.05). The dendritic spine density further decreased from 15 to 20 months while it remained unchanged in age-matched wild type control mice (Fig. 1C and 1D). The threshold of 50 µm was used since previous studies in different AD mouse models have shown that the dendritic spine density is predominantly reduced within a radial distance of 50 µm around amyloid plaques [26]–[28]. In conclusion, spine loss in the hippocampus was not only apparent in proximity to amyloid plaques. It also occurred in areas distant from plaques.

### Dendritic spine loss in the somatosensory cortex

**Figure 2:**
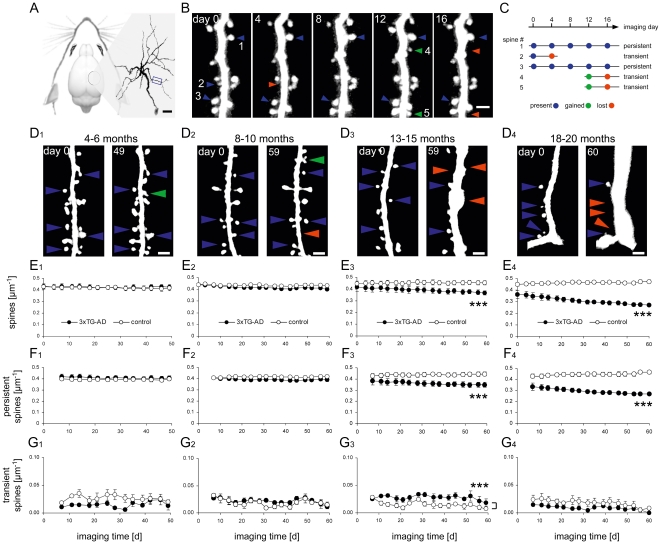
Dendritic spine density progressively declines in the somatosensory cortex from 13 months on. (A) A cranial window was implanted over the somatosensory cortex to perform long-term two-photon *in vivo* imaging of neurons, dendrites, and dendritic spines. (B) High-resolution time-lapse images of a dendritic region (blue box in A) over 16 days. Present spines (blue arrows), lost spines (red arrows), and gained spines (green arrows) are exemplarily marked. (C) Classification of the 5 spines marked in B in categories of persistent (lifetime ≥8 days) and transient spines (lifetime <8 days). (D) Start and end point images of a 49-60 day *in vivo* imaging period of dendrites and dendritic spines in the somatosensory cortex of 4–6 (D_1_), 8–10 (D_2_), 13–15 (D_3_), and 18–20 (D_4_) months old 3xTg-AD mice. Color code of arrows as in B. (E–G) Dendritic spines imaged in 4–6 (_1_), 8–10 (_2_), 13–15 (_3_), and 18–20 (_4_) months old 3xTg-AD mice and age-matched non-AD transgenic control mice over a 49–60 day *in vivo* imaging period. (E) Dendritic spine density, (F) density of persistent dendritic spines, (G) density of transient dendritic spines. Each circle indicates one imaging time point ±S.E.M. (*** P<0.001, repeated measures ANOVA (E and F) or Wilcoxon Rank Sum Test (G), n = 4 mice per group). Scale bars: 20 µm (A), 2 µm (B and D);

To analyze dendritic spine loss in the absence of amyloid plaques in more detail, we monitored dendritic spines in the somatosensory cortex where no amyloid plaques are present at any age (Fig. S1B). By long-term two-photon *in vivo* imaging (Fig. 2A–C), the fate of individual dendritic spines was analyzed in four age groups (4–6, 8–10, 13–15, and 18–20 months) in 3xTg-AD mice and age-matched non-AD transgenic control mice (n = 4 mice per group) for up to 60 days (in total n = 100,642 spines).

Dendritic spine density did not change over time (Fig. 2D_1_, 2D_2_, 2E_1_, 2E_2_) in 4–6 and 8–10 months-old 3xTg-AD mice, when only soluble Aβ, but neither hyperphosphorylated tau nor amyloid plaques are present in the somatosensory cortex (Fig. S1). In these age groups, dendritic spine density remained at a level comparable to age-matched wild type controls (Fig. 2D_1_, 2D_2_, 2E_1_, 2E_2_). At 13-15 months of age, when both soluble Aβ and hyperphosphorylated tau accumulate in the somatosensory cortex (Fig. S1), dendritic spine density significantly decreased over time by 1.84±0.84 spines day^−1^ mm^−1^ (Fig. 2D_3_, 2E_3_; 0.42±0.04 µm^−1^ to 0.36±0.02 µm^−1^, P<0.001). The decline was even stronger in 18–20 month-old 3xTg-AD mice and progressed with 4.25±0.79 spines day^−1^ mm^−1^ (Fig. 2D_4_, 2E_4_; 0.36±0.03 µm^−1^ to 0.27±0.01 µm^−1^, P<0.001). At 13–15 and 18–20 months of age, the spine density of wild type mice did not decrease over time (Fig. 2D_3_, 2D_4_, 2E_3_, 2E_4_).

In addition to dendritic spine density, the experimental design enabled us to analyze the fate of functional synapses over time. Dendritic spines with a lifetime of more than eight days were classified as “persistent” spines (Fig. 2B and 2C). It has been shown that the majority of persistent spines predominantly forms functional synapses with presynaptic boutons [52]. In contrast, short-lived “transient” dendritic spines, with a lifetime of less than eight days, primarily represent non-functional synapses [39], [44], [51], [52]. Between 4–6 and 8–10 months of age, when the spine density remained constant, neither the persistent nor the transient spine density was altered (Fig. 2F_1_, 2F_2_, 2G_1_, 2G_2_). This changed at 13–15 months, when specifically persistent spines were lost (Fig. 2F_3_). Interestingly, the density of transient spines was significantly increased during this time-period, compensating at least partially for the loss of persistent spines (Fig. 2G_3_; 0.028±0.009 µm^−1^ to 0.015±0.007 µm^−1^, P<0.001). This compensatory increase in the density of transient spines was not apparent at 18–20 months (Fig. 2G_4_), resulting in an even more pronounced loss of spines at this age (Fig. 2E_4_).

### Dendritic dystrophy coincides with dendritic spine loss in the somatosensory cortex

**Figure 3:**
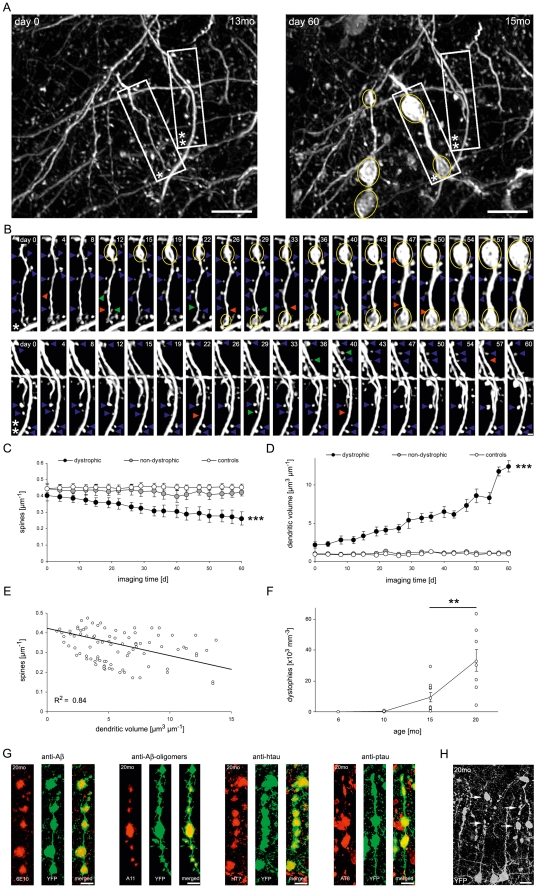
Dendritic spine density is reduced exclusively at dystrophic, soluble Aβ and hyperphosphorylated tau accumulating dendrites. (A, B) Time-lapse images of dystrophic and non-dystrophic dendrites over 60 days. Dendritic dystrophies (yellow circles), stable (blue arrows), lost (red arrows), and gained dendritic spines (green arrows) at corresponding time-points are labeled. (C, D) Changes in dendritic spine density (C) and dendritic volume (D) over 60 days at dystrophic, non-dystrophic and control dendrites in 13–20 month-old mice. Note that the dendritic spine density of dystrophic dendrites significantly decreased over time, while the mean dendritic volume of the same dendrites significantly increased (*** P<0.001, repeated measures ANOVA, n = 4 mice per group). Each circle represents one imaging time-point. (E) In 3xTg-AD mice, the dendritic volume and spine density are inversely correlated (R^2^ = 0.84, P<0.001, n = 4 mice), indicating a relationship between both events. (F) The mean number of dystrophic dendrites per volume significantly increased from 15 to 20 months of age (** P<0.01, Student's t-test, n = 8–10 mice per age group). (G) Dendritic dystrophies in the somatosensory cortex were labeled positive for antibodies 6E10, A11, HT7, and AT8 via immunofluorescence staining. Co-localization (yellow) of each antibody (red) and YFP (green) in merged images. (H) Cortical section of a 20 month-old 3xTg-AD mouse expressing YFP in cortical neurons that has not previously been imaged *in vivo*. Dystrophic dendrites (white arrows) are abundant in cortical layers 1–3. Error bars: ±S.E.M. Scale bars: 10 µm (A, G, H) 2 µm (B),

At the same time when we observed a decline in dendritic spine density in the somatosensory cortex of 13–15 and 18–20 months-old 3xTg-AD mice, we noticed that some dendrites showed a dystrophic volume increase over time (Fig. 3A and 3B). Figures 3A and 3B exemplarily illustrate a dendrite that became dystrophic and lost spines over 60 days compared to a neighboring dendrite that neither exhibited dystrophic swellings nor spine loss (see also Video S1). The analysis of spines at dystrophic and non-dystrophic dendrites revealed that dystrophic dendrites showed a significant decline in spine density over time (Fig. 3C; 0.40±0.03 µm^−1^ to 0.26±0.04 µm^−1^, P<0.001). Dendrites of wild type control mice displayed unchanged spine densities and dystrophic changes were never observed (Fig. 3C). Interestingly, the volume of dystrophic dendrites did not stay at a constant level over time, but significantly increased (P<0.001), indicating a progressing pathology (Fig. 3D). Moreover, a strong correlation was identified between a reduction of dendritic spine density and an increase in dendritic volume (Fig. 3E; R^2^ = 0.84, P<0.001). Additionally, as shown above, the spine density was unaffected before the first dystrophies emerged at 13 months (Fig. 2E_1_ and 2E_2_). From that age on, the number of dystrophies significantly increased from 9.58±3.05×10^3^ mm^−3^ at 15 months to 33.23±7.02×10^3^ mm^−3^ at 20 months (Fig. 3F; P = 0.004), indicating a progression with age.

In order to clarify if soluble Aβ and tau were present in dystrophic dendrites, we performed immunohistochemistry with antibodies 6E10, A11, HT7, and AT8 on fixed slices (Fig. 3G). Indeed, soluble Aβ and hyperphosphorylated tau protein were abundant in all analyzed dystrophic dendrites (n = 2,305 dystrophies in n = 13 mice). This strongly indicates a relationship between intracellular Aβ and hyperphosphorylated tau accumulation on one side and spine loss at dystrophic dendrites on the other.

We can exclude the possibility that dendritic dystrophy was caused by cranial window surgery and laser-induced phototoxicity, since neighboring dendrites that were imaged with the identical parameters did not exhibit any dystrophic changes (Fig. 3A and 3B and Video S1). In addition, dystrophic dendrites were also apparent in cortical slices of 3xTg-AD mice that underwent neither cranial window surgery, nor two-photon *in vivo* imaging (Fig. 3G and 3H).

### Early layer III neuron loss in the somatosensory cortex

**Figure 4:**
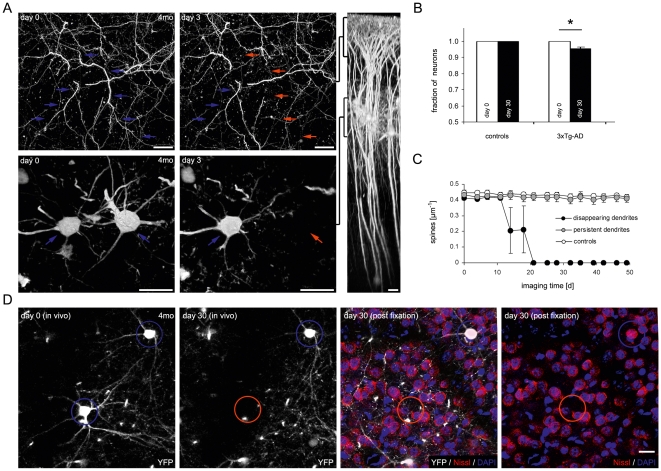
Early layer III neuron loss leads to spine loss. (A) Two-photon *in vivo* images of the identical cortical region of a 4 months old 3xTg-AD mouse showing a selective disappearance of an apical dendrite in layer 1–2 (red arrows) and the corresponding layer III neuron after 3 days, while a neighboring dendrite and neuron persisted (blue arrows). Scale bars: 20 µm. (B) Neuron loss in a 30 day interval was exclusively detected in 3xTg-AD mice compared to control mice (* P<0.05, Student's t-test, n = 1,200 neurons). Error bars indicate +S.E.M. (C) Mean dendritic spine density of disappearing dendrites remained unchanged before the loss of the neuron. Dendritic spine density remained at a level comparable to the spine density of persistent dendrites of the same 4–6 months old 3xTg-AD mice and controls (n = 10 dendrites per group). Error bars indicate ±S.E.M. (D) Two-photon *in vivo* images of the same layer III neurons in a 4 months old 3xTg-AD mouse and 30 days later. A single neuron disappeared after 30 days (red circle) while a neighboring YFP positive neuron persisted (blue circle). After the neuron loss occurred, the brain was paraformaldehyde fixed, cut in 100 µm thick sections, and the nuclei were labeled with DAPI while neurons were selectively labeled with Nissl-Red. Neither DAPI, nor Nissl staining was detected at the position where neuron loss was previously detected in the *in vivo* image (red circle) while the persisting neuron was labeled with DAPI and Nissl (blue circle). Scale bars: 20 µm.

While we analyzed dendritic spines on apical dendrites of layer III and layer V cortical neurons in 3xTg-AD mice, we occasionally observed dendrites disappearing from one imaging time point to the next (Fig. 4A). When we followed the disappearing dendrite to the corresponding layer III neuron in previous images, we realized that the neuron had in fact disappeared along with the dendrite (Fig. 4A). Neighboring neurons and dendrites persisted over the entire imaging period (Fig. 4A), whereas the disappeared neurons and dendrites did not reappear in subsequent imaging sessions.

Based on these observations, layer III neuron loss was systematically analyzed by two-photon *in vivo* imaging stereology as previously described [45]. Therefore, identical YFP expressing layer III neurons were imaged and counted at day 0 and 30 days later in 3xTg-AD and non-transgenic mice. Neuron loss manifested only in 3xTg-AD mice, whereas not a single neuron loss was detected in non-AD transgenic control mice (Fig. 4B; decrease of 4.6±1.1% vs. 0.0±0.0% respectively; P = 0.012, Student's t-test; n = 1,200 neurons imaged in a total volume of 2.22 mm^3^ in n = 18 mice). Layer III neuron loss was apparent at 4–6 months of age when oligomeric Aβ accumulates in 3xTg-AD mice [53].

Importantly, before dendrites disappeared due to neuron loss their spine density was comparable to the spine density of dendrites that persisted (Fig. 4C; 0.40±0.01 µm^−1^ vs. 0.43±0.02 µm^−1^ respectively; n = 10 dendrites per group). This indicates that the dendritic spine density was not altered before the neuron was lost and therefore spine loss did not precede layer III neuron loss. Obviously, when a neuron was lost all dendrites and spines that protruded from this neuron were lost, too.

To determine if the disappearance of neurons was based on a loss of YFP expression or if the neuron itself was lost, we performed post-mortem nuclear DAPI- and neuronal Nissl-staining of the same area where neuron loss has previously been detected by two-photon *in vivo* imaging. This experimental approach has previously been described in detail [45]. As expected, the lost neurons were not labeled positive for DAPI or Nissl compared to neighboring neurons that were still present (Fig. 4D). This provides strong evidence, that these neurons were in fact lost and did not just loose YFP expression.

## Discussion

It has previously been shown that Aβ oligomer administration [23], [24] and virally induced overexpression of mutated APP [22], [25] led to a reduced dendritic spine density in hippocampal slice cultures. Further evidence from AD mouse models shows that a decline in the density of the synaptic marker synaptophysin and in dendritic spine density have also been detected at a time, when only soluble Aβ but no amyloid plaques were present [15]–[18].

Our working hypothesis was to determine if this early decline in dendritic spine density is also apparent in 3xTg-AD mice [49] where cognitive decline [54] and accumulation of oligomeric Aβ [53] occurs as early as 4–6 months of age before amyloid plaques and hyperphosphorylated tau are present. To our surprise, at those early stages, loss of dendritic spines was only observed as a consequence of layer III neuron loss. This indicates that soluble Aβ, which accumulates at this age [45], [53], is toxic at least for a subset of cortical neurons. Most importantly, in 3xTg-AD mice we did not find any evidence that soluble Aβ leads to dendritic spine loss at intact dendrites as anticipated by previous studies [16], [17], [21]–[25]. How can this discrepancy be explained? One possibility is that in 3xTg-AD mice the soluble Aβ concentrations at dendritic spines are significantly lower than the concentrations that led to dendritic spine loss in the previous *in vitro* studies [21]–[25]. In addition, the spatio-temporal distribution of soluble Aβ might be different in 3xTg-AD compared to other AD mouse models that show an early decline in spine density [16], [17].

In 3xTg-AD mice, a decline in the density of dendritic spines of individual dendrites could first be observed at the age of 13 months. Hereby, the dendritic spine density was reduced in close proximity to amyloid plaques. This finding is in line with observations from other mouse models [26]–[31]. In addition, 3xTg-AD mice show a strong decline in spine density also distant to amyloid plaques. Interestingly, spine density was exclusively reduced at dystrophic dendrites, where both oligomeric Aβ and hyperphosphorylated tau were intracellularly abundant.

Collectively, three spatio-temporally independent events lead to a net loss of dendritic spines in 3xTg-AD mice. First, layer III neuron loss occurs early at the time when soluble Aβ accumulates. Consequently, dendritic spines of these neurons get lost. Second, from 13 months on, at a time when amyloid plaques are abundant, the dendritic spine density declines in proximity to amyloid plaques. Third, starting at 13 months, a reduction in dendritic spine density manifests also in areas apart from amyloid plaques. This plaque independent spine loss occurs exclusively at dystrophic dendrites that accumulate both Aβ oligomers and hyperphosphorylated tau intracellularly. Taken together, we identified three independent events that contribute to a loss of dendritic spines in 3xTg-AD. Each of these events can individually lead to dendritic spine loss. This implies that AD therapies which are only directed at one of these causes probably fall short to counteract the full spectrum of dendritic spine loss in AD.

## Supporting Information

Figure S1 **Aβ and tau pathology in 3xTg-AD mice.** (A-C) Immunofluorescence images of hippocampal and cortical slices of 6, 10, 15, and 20 month-old 3xTg-AD mice stained with 6E10 antibody (A), methoxy-X04 which labels fibrillar aggregates like A**β** plaques (arrows) or neurofibrillary tangles (B). AT8 antibody binds to hyperphosphorylated tau (C). Scale bars: 20 µm (hippocampus) 50 µm (cortex) (TIF)

Video S1 **Dendritic dystrophy in 3xTg-AD mice.** In 13 months old 3xTg-AD mice, some dendrites started to become dystrophic while neighboring dendrites showed a normal volume over the whole imaging period of 60 days. Scale bar: 10 µm (AVI)
