# Supplementary figures and images for: Multiple Events Lead to Dendritic Spine Loss in Triple Transgenic Alzheimer's Disease Mice
Source: PLoS One. 2010 Nov 16;5(11):e15477. doi: 10.1371/journal.pone.0015477 (PMC2982845; doi:10.1371/journal.pone.0015477)

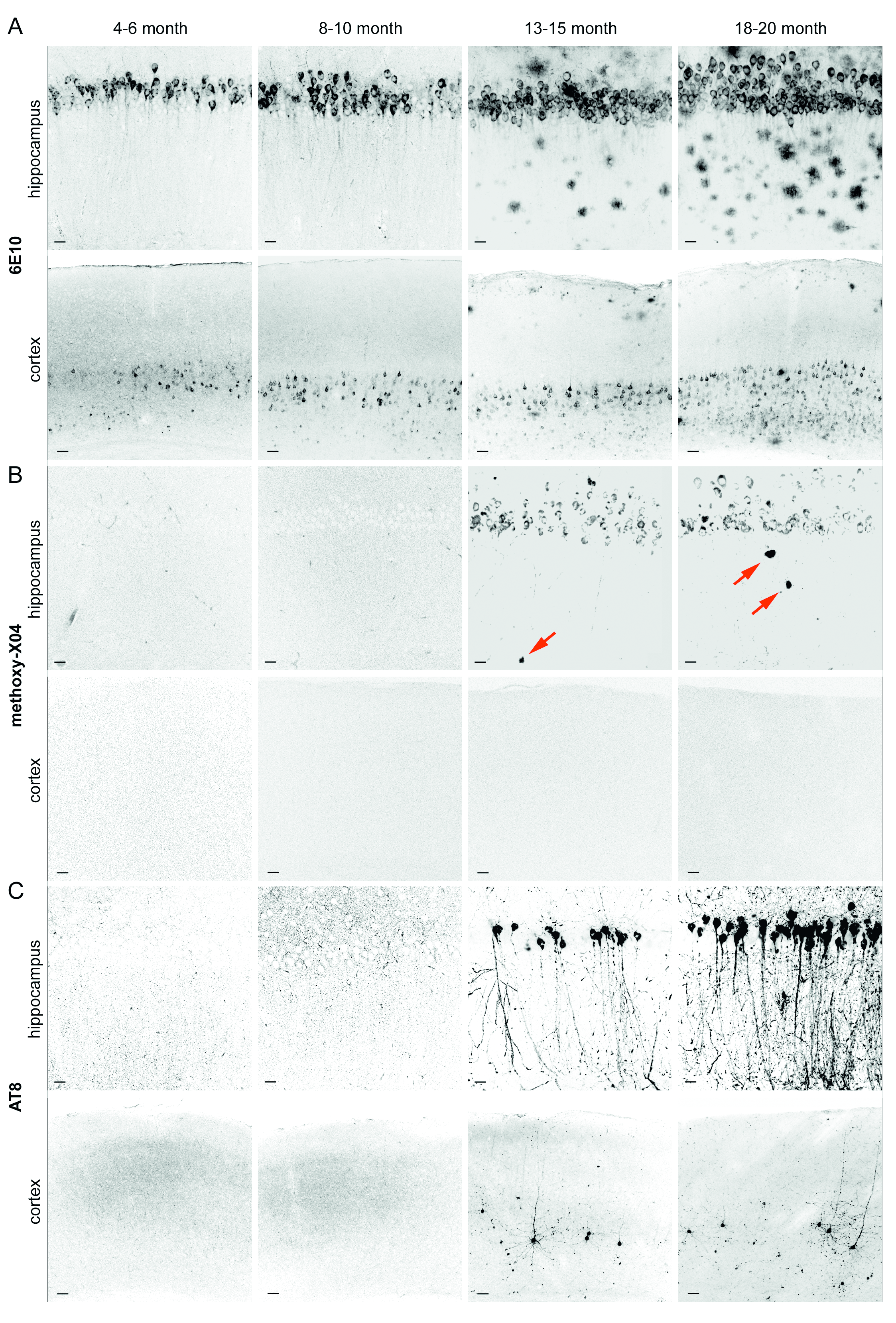

Supplement: Figure S1 — Aβ and tau pathology in 3xTg-AD mice. (A-C) Immunofluorescence images of hippocampal and cortical slices of 6, 10, 15, and 20 month-old 3xTg-AD mice stained with 6E10 antibody (A), methoxy-X04 which labels fibrillar aggregates like Aβ plaques (arrows) or neurofibrillary tangles (B). AT8 antibody binds to hyperphosphorylated tau (C). Scale bars: 20 µm (hippocampus) 50 µm (cortex) (TIF) [file pone.0015477.s001.tif]
